# Racial Disparities in Methylation of *NRF1*, *FTO*, and *LEPR* Gene in Childhood Obesity

**DOI:** 10.3390/genes13112030

**Published:** 2022-11-04

**Authors:** Priyadarshni Patel, Vaithinathan Selvaraju, Jeganathan Ramesh Babu, Xu Wang, Thangiah Geetha

**Affiliations:** 1 Department of Nutritional Sciences, Auburn University, Auburn, AL 36849, USA; 2 Boshell Metabolic Diseases and Diabetes Program, Auburn University, Auburn, AL 36849, USA; 3 Alabama Agricultural Experiment Station, Auburn University, Auburn, AL 36849, USA; 4 Department of Pathobiology, College of Veterinary Medicine, Auburn University, Auburn, AL 36849, USA; 5 HudsonAlpha Institute for Biotechnology, Huntsville, AL 35806, USA

**Keywords:** childhood obesity, DNA methylation, epigenetics, health disparities, *NRF1*, *FTO*, *LEPR*

## Abstract

Childhood obesity has affected the health of millions of children around the world despite vigorous efforts by health experts. The obesity epidemic in the United States has disproportionately afflicted certain racial and ethnic minority groups. African American children are more likely than other children to have obesity-related risk factors such as hyperlipidemia, diabetes, cardiovascular disease, and coronavirus disease (COVID-19). For the reduction in obesity-related health inequalities to be successful, it is essential to identify the variables affecting various groups. A notable advancement in epigenetic biology has been made over the past decade. Epigenetic changes like DNA methylation impact on many genes associated with obesity. Here, we evaluated the DNA methylation levels of the genes *NRF1*, *FTO*, and *LEPR* from the saliva of children using real-time quantitative PCR-based multiplex MethyLight technology. *ALU* was used as a reference gene, and the Percent of Methylated Reference (PMR) was calculated for each sample. European American children showed a significant increase in PMR of *NRF1* and *FTO* in overweight/obese participants compared to normal weight, but not in African American children. After adjusting for maternal education and annual family income by regression analysis, the PMR of *NRF1* and *FTO* was significantly associated with BMI *z*-score only in European American children. While for the gene *LEPR*, African American children had higher methylation in normal weight participants as compared to overweight/obese and no methylation difference in European American children. The PMR of LEPR was significantly negative associated with the obesity measures only in African American children. These findings contribute to a race-specific link between *NRF1*, *FTO*, and *LEPR* gene methylation and childhood obesity.

## 1. Introduction

Type 2 diabetes, heart disease, dyslipidemia, hypertension, hepatic steatosis, and metabolic syndrome are among comorbidities that can arise because of inappropriate or excessive fat deposition in the body [1]. The frequency of childhood obesity has been rising alarmingly over the past 40 years. In the United States, childhood obesity remains an epidemic affecting the health of millions of children. According to the Centers for Disease Control and Prevention (CDC), the prevalence of obesity was 12.7% of children aged 2 to 5 years, 20.7% of children aged 6 to 11 years, and 22.2% of children aged 12 to 19 years were obese in 2017–2020 [2]. Alabama is fifth highest in the nation for the prevalence of obesity with around 22% of children and adolescents aged 10 to 17 years being overweight and obese [3]. When compared to other ethnic groups, some have a greater prevalence of obesity, as is the case with African American children, where obesity continues to be a major public health issue [4]. To successfully lower obesity-related health inequalities it is essential to identify the variables that affect various populations [5].

The major factors contributing to the increase in the occurrence of obesity might be due to a sedentary lifestyle [6], lack of nutritious food [7], environmental factors [8], socioeconomic status [9], and genetic factors [10]. Even while genetic factors influence a person’s susceptibility to weight gain and obesity, the genetic variations discovered only explain a small percentage of the variation. This has raised curiosity about the potential function of epigenetics as a modulator of gene-environment interactions in the development of obesity and its associated comorbidities [11,12]. Epigenetics can be defined as “the study of changes in organisms caused by modification of gene expression rather than alteration of the genetic code itself” [13]. Diet, obesity, physical exercise, cigarette smoking, alcohol use, environmental contaminants, and psychological stress have all been identified as lifestyle factors that may alter epigenetic patterns [14,15]. Instead of affecting the underlying DNA sequences, epigenetic changes alter gene expressions without changing DNA sequences [16]. The most crucial epigenetic mechanisms in regulating gene activity are DNA methylation, histone modifications, and non-coding RNAs [16].

The most widely studied epigenetic mark in the human genome in terms of controlling gene expression is DNA methylation, which is an epigenetic process involving the covalent attachment of a methyl group (-CH3) onto the fifth position of cytosine, resulting in 5-methylcytosine. Although methylation in mammals is mostly limited to cytosine connected to guanine by a phosphate (CpG site), non-CpG sites (CHG and CHH, where H = A, C, or T) can also be methylated [17,18]. Dietary, pharmacological, and physical factors influence epigenetic modifications, resulting in a change in gene expression profile [19]. Individual disparities in susceptibility to obesity and other metabolic illnesses may be caused by variances in DNA methylation patterns [20]. Different research studies such as the candidate gene approach genome-wide analysis have shown a link between obesity and methylation at specific genes [21,22].

In the present study, we focused on the three genes *NRF1*, *FTO*, and *LEPR*. Nuclear respiratory factor 1 (*NRF1*) is a CNC (cap-’n’-collar) family transcription factor containing a leucine zipper in the basic region. It has a link to the innate immune response, which controls brown adipose tissue’s thermogenic adaption, adipocyte inflammation, and cytokine production. Studies have revealed that *NRF1* increases insulin resistance [23,24,25]. The role of *NRF1* in pathophysiology is still unknown. The potential link between *FTO* and BMI was first discovered in 2007 in a genome-wide association study [26]. A similar association between the *FTO* variants and body weight was found in 13 cohorts of 38,759 Britons, Finns, and Italians. It is regarded as the first and most significantly linked gene with obesity in several populations across various nations [27]. *FTO* is expressed in the nucleus of every cell in the human body. The gene controls energy balance and eating behavior in the hypothalamus and its arcuate, paraventricular, dorsomedial, and ventromedial nuclei [25,27,28]. Notably, both in vivo and in vitro data showed that *FTO* could sense nutritional status and respond to appetite and food intake, either directly by adipocyte or indirectly by hypothalamus-controlled neurologic circuitry, offering some insight into the complicated biological functions of *FTO* [29]. The gene *LEPR* encodes the receptor for leptin (*LEP*), a protein hormone mostly produced by adipose tissue. An adipocyte-derived cytokine called *LEP* interacts with *LEPR* to control satiety and energy expenditure. The brain’s hypothalamus region is home to leptin receptors, which are crucial for controlling appetite and preventing the onset of obesity [30,31].

Epigenetic processes have a great chance of explaining the molecular routes through which different health inequalities impact obesity. Minority and disadvantaged groups continue to be disproportionately affected by health disparities in the United States, resulting in huge disparities in morbidity and mortality. Despite increased access to health care, African Americans have continuously poorer health outcomes than white Americans [8]. When compared to whites, African Americans had much higher rates of obesity, hypertension, and death from cardiovascular disease [32]. Therefore, the main aim of this study was to look at the racial differences in the PMR of the genes *NRF1*, *FTO*, and *LEPR* amongst normal weight (NW) and overweight (OW)/obese (OB) children from two racial groups European American (EA) and African American (AA).

## 2. Materials and Methods

### 2.1. Study Participants

In all, 113 research participants between the ages of 6 and 10 (8.57 ± 0.13) years were recruited from Lee and Macon counties in Alabama. Following a preliminary phone interview with the parents, children with a history of diabetes or cardiovascular disease were eliminated. Parents and participants both provided their written approval. Parents brought their kids to Auburn University so that their anthropometric measurements and saliva samples were collected. The study was approved by the institutional review board of Auburn University (Protocol # 17-364 MR 1709).

### 2.2. Anthropometric Measurements

The World Health Organization (WHO) guidelines were followed for taking the participants’ anthropometric measures. The children’s body weight was measured on a Tanita digital scale to the closest 4 ounces, without shoes, and in light clothes. The height was measured with a calibrated scale that was connected to a stadiometer, and the accuracy was 0.1 cm. Children have different ratios of bone, muscle, and fat as they become older. Therefore, the BMI *z*-score (Body Mass Index) is a more accurate way to assess a developing child’s weight than the BMI alone. Using the SPSS macro, the BMI *z*-score was computed using the WHO growth references adjusted for age and sex [33]. The classification for children was based on the CDC standards as underweight (<5th percentile), normal weight (≥5th percentile to ≤85th percentile), overweight (≥85th percentile to ≤95th percentile), and obese (≥95th percentile). The waist circumference was measured at the midpoint between the lower ribs and iliac crest using a non-elastic tape to the nearest 0.1 cm. R macro package was used to calculate the *z*-score of waist circumference (WC) and waist:height ratio (WHtR) [34].

### 2.3. Isolation of Salivary DNA

Saliva was collected using the Oragene Geno-Tek saliva collection kit (Catalog # OGR-500; Ottawa, ON, Canada). As per the manufacturer’s instructions, saliva samples were incubated in the water bath at 50 °C for 3 h. A 500 µL aliquot was used to isolate DNA using the PrepIT.L2P DNA isolation kit (Catalog # PT-L2P-5; DNA genotek, Ottawa, ON, Canada). Each sample was labeled and stored at −20 °C until further use. Isolated DNA was quantified, and the quality was checked using a NanoDrop ND-1000 spectrophotometer (Thermo Fisher Scientific, Inc., Wilmington, DE, USA), which specifically measured the double-stranded DNA.

### 2.4. Bisulfite Conversion

The bisulfite conversion of DNA leads to the deamination of unmethylated cytosines, which are converted to uracil and subsequently to thymine in the subsequent PCR, while the cytosines that are methylated remain unchanged and this makes it possible to analyze the methylation pattern of the DNA sequence. The bisulfite conversion of non-methylated cytosines to uracil was done using the EpiTect Fast DNA Bisulfite kit (Catalog # 59720; QIAGEN, Germany). The reaction consisted of bisulfite solution, buffer, 1 ng of gDNA, and RNase-free water to make the volume up to 140 µL. Thermo Fisher Quantstudio 3 was used to run the thermal cycle under the following condition: 95 °C for 5 min, 60 °C for 10 min, 95 °C for 5 min, 60 °C for 10 min, and 20 °C for 10 min. After the bisulfite conversion, DNA was cleaned up by following the protocol provided by the manufacturer. The product was eluted using 15 µL of elution buffer and stored at −80 °C until used. Once the bisulfite conversion is done, the gDNA behaves like RNA, further quantified using nanodrop, which specifically measured the ssRNA. All samples were normalized to 5 ng/µL per nanodrop quantification before the multiplex MethyLight RT-PCR reaction.

### 2.5. Multiplex MethyLight Primer and Probe Design

The Multiplex MethyLight assay was carried out using two primers and a TaqMan probe for each gene. Primers and Probes were specifically designed for bisulfite-converted DNA, which can amplify methylated DNA using Beacon Designer 8.21 (Premier Biosoft International, Palo Alto, CA, USA) for three genes: *NRF1*, *FTO*, *LEPR*, and *ALU*. *ALU* was used as a reference gene in every well to normalize the input DNA. The primer and probe sequences for all the genes are listed in Table 1.

### 2.6. MethyLight RT-PCR Reaction

MethyLight is a TaqMan-based qPCR method that is extremely sensitive and depends on the hybridization and cleavage of probes that are intended to target the CpG of interest [35]. Using Quantstudio 3, fluorescence-based real-time quantitative PCR was used to amplify bisulfite converted DNA after sodium bisulfite conversion in a 96-well plate. The PCR amplification was carried out using the Epitect MethyLight PCR + ROX vial kit (Qiagen GmbH; catalog # 59496) according to the manufacturer’s instructions. The final volume of the reaction was 20 µL, and it contained 400 nM of each primer, 250 nM of each probe, 10 µL of the MethyLight master mix (HotStar Taq Plus DNA Polymerase, Epitect Probe PCR Buffer, dNTP mix- dATP, dCTP, dGTP, dTTP), 0.4 µL ROX dye, RNase free water and 10 ng of bisulfite converted DNA. There were 45 cycles of the following cycling conditions: 95 °C for 5 min, 95 °C for 15 s, and 60 °C for 60 s. In addition to gDNA and the No Template Control (NTC), universally methylated and non-methylated standards were employed as a control in each plate. Additionally, commercially available human bisulfite-converted DNA (EpiTect PCR Control DNA; catalog # 59655; Qiagen GmbH) was utilized as a completely methylated control for the computation of the PMR. The PMR is a relative value of methylation in each sample as compared to the fully methylated control. PCR primers surrounding an oligonucleotide probe with a 5′ fluorescent reporter dye and a 3′ quencher dye are used to amplify bisulfite-converted genomic DNA. Taq DNA polymerase’s 5′ to 3′ nuclease activity cleaves the probe and releases the reporter, whose fluorescence can be measured. The PCR amplification produces a fluorescent signal proportional to the PCR product created after crossing a fluorescence detection threshold. The cycle number at which the fluorescent signal passes a threshold in the exponential phase of the PCR reaction may be used to calculate the initial template amount.

### 2.7. Statistical Analysis

The PMR of genes *NRF1*, *FTO*, and *LEPR* for each sample was calculated using the formula PMR = 100 × 2^−ΔΔCt^, where ΔΔCt = [ΔCt of sample—ΔCt of universal methylated DNA]. The calculations for PMR values were done using Microsoft Excel. IBM SPSS Statistics 25.0 was used to do independent samples t-test to see the difference between the PMR of NW and OW/OB participants. Normality tests were performed to confirm the normal distribution of the data. To analyze the effect of maternal education and annual family income on the PMR of *NRF1*, *FTO*, and *LEPR* hierarchical regression was performed. The value *p* < 0.05 was considered to indicate a statistically significant difference. Linear regression analysis was performed to determine the association between the log-transformed PMR values of each gene and BMI *z*-score, WC *z*-score, and WHtR *z*-score. A Receiver Operating Characteristics (ROC) curve was generated using the PMR values of *NRF1*, *FTO*, *LEPR*, and appropriate cut-off values for BMI categories and race were calculated for the genes using the SPSS software.

## 3. Results

The study participants included 60 NW and 53 OW/OB children aged 6 to 10 years. Table 2 shows the general characteristics of the study population. The mean age and height were not statistically different amongst the groups. As expected, when compared to NW children, OW/OB children had significantly higher anthropometric characteristics including BMI *z*-score, WC *z*-score, and WtHR *z*-score. Table 3 shows the general characteristics of the family income and maternal education of the study population, where a higher percentage (55.6%) of EA had a family income greater than USD 75,000 while only 20% of AA had that income. Moreover, the percentage of AA having income less than USD 25,000 was 66%, while that in EA was only 4.8%. For maternal education, a higher percentage (46%) of AA had education up to high school or less, EA only 9.8%. At the higher end, 42.9% of EA had a graduate degree compared to AA with 18% only.

**Figure 1:**
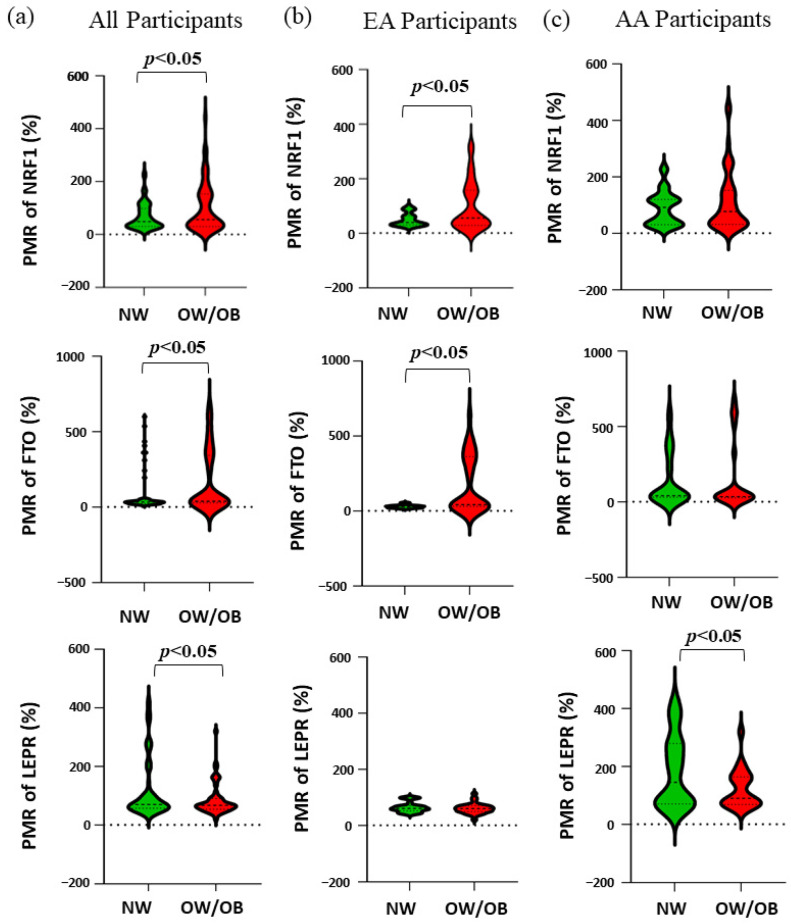
The violin plots show the distribution of the PMR values between NW and OW/OB children. (**a**) The PMR of *NRF1* between NW and OW/OB children in all, EA, and AA participants. (**b**) The PMR of *FTO* between NW and OW/OB children in all, EA, and AA participants. (**c**) The PMR of *LEPR* between NW and OW/OB children in all, EA, and AA participants. *p* < 0.05 is considered statistically significant.

Table 4 and Figure 1 show the race-specific PMR of *NRF1*, *FTO*, and *LEPR* genes. In all participants, NW children had significantly lower methylation for the genes *NRF1* (*p* = 0.018) and *FTO* (*p* = 0.010), while significantly higher methylation in gene *LEPR* (*p* = 0.025) compared to OW/OB children. Further, by separating the participants by their race, in EA children our results showed a similar trend for the genes *NRF1* (*p* = 0.002) and *FTO* (*p* = 0.001) where NW children had less methylation but there was no significant difference observed in the methylation of the gene *LEPR*. Interestingly, for AA no significant difference in the methylation of the genes *NRF1* and *FTO* was found since AA NW children had higher methylation than EA NW children, while for the gene *LEPR*, NW children had higher methylation compared to OW/OB (*p* = 0.046).

**Figure 2:**
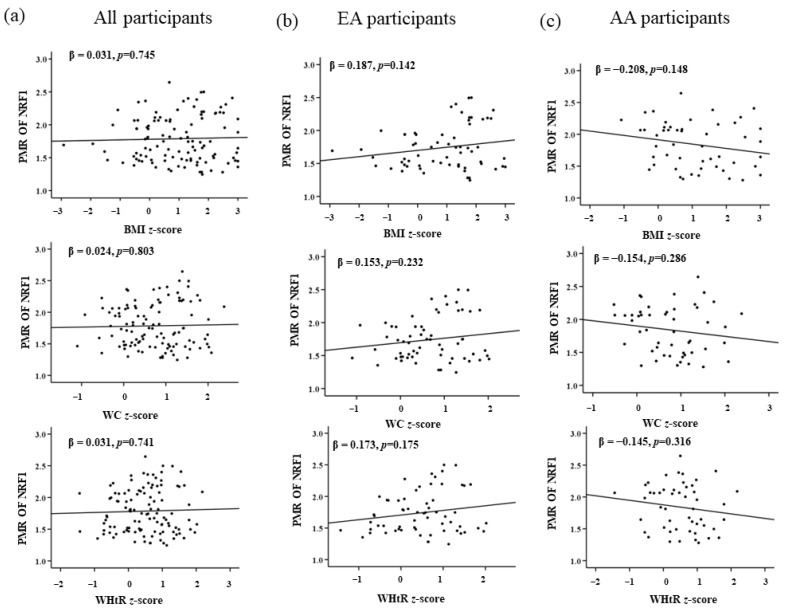
Association of *NRF1* with childhood obesity markers of (**a**) all, (**b**) EA, and (**c**) AA participants. *p* < 0.05 is considered statistically significant.

**Figure 3:**
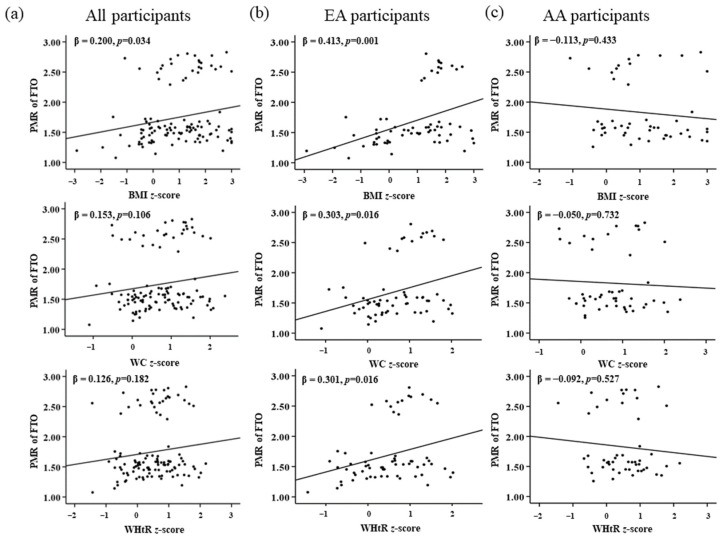
Association of *FTO* with childhood obesity markers of (**a**) all, (**b**) EA, and (**c**) AA participants. *p* < 0.05 is considered statistically significant.

**Figure 4:**
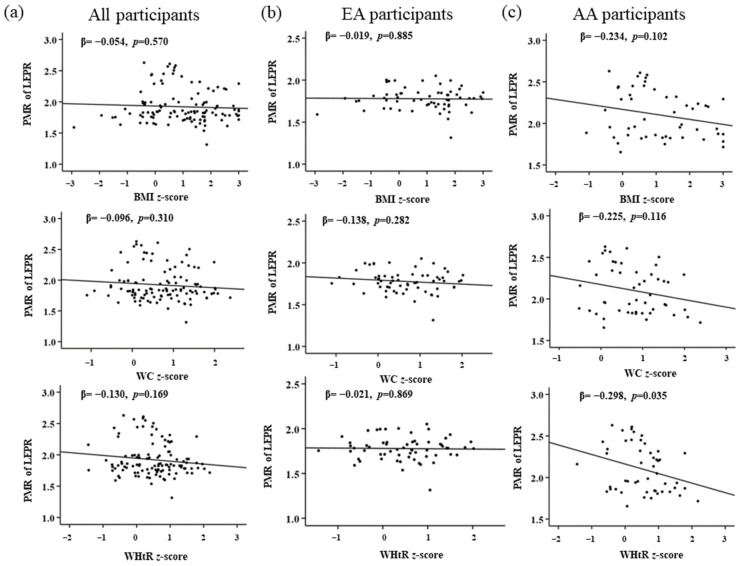
Association of *LEPR* with childhood obesity markers of (**a**) all, (**b**) EA, and (**c**) AA participants. *p* < 0.05 is considered statistically significant.

A multinominal linear regression between PMR of *NRF1*, *FTO*, and *LEPR* with the obesity measures (BMI *z*-score, WC *z*-score, and WHtR *z*-score) after adjusting with the covariates (family income and maternal education) demonstrated a significant positive correlation between PMR of *NRF1* in EA participants with BMI *z*-score in the adjusted and unadjusted model. Meanwhile, AA participants showed a negative correlation between PMR of *NRF1* and obesity measures with no statistical significance (Table 5). Similar calculations were performed for PMR of *FTO* and obesity measures. For EA participants, a significant positive correlation was observed with the BMI *z*-score, WC *z*-score, and WHtR *z*-score for both adjusted and unadjusted models. While AA participants, there is a negative correlation with no statistical significance of *FTO* with obesity measures (Table 6). For the gene *LEPR*, total participants and EA showed no significant association while in AA, BMI *z*-score, and WtHR *z*-score had a significant negative association with *LEPR* for both adjusted and unadjusted models. Interestingly, we could see a significant negative association between the WC *z*-score and PMR of *LEPR* only in the adjusted model (Table 7). Maternal education and family income did not affect the PMR of *NRF1* and *FTO*. Nonetheless, some correlation was seen with PMR of *LEPR* and WC *z*-score in AA children, which could be affected by family income and maternal education. Graphical representations from linear regression analysis for all three genes are represented in Figure 2, Figure 3 and Figure 4.

**Figure 5:**
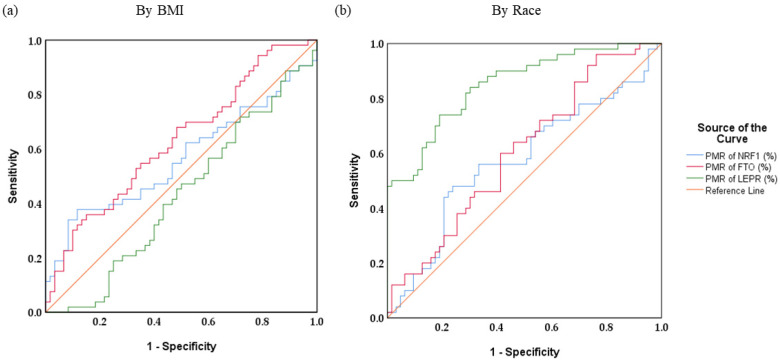
Receiver operating characteristic (ROC) curves for *NRF1*, *FTO*, and *LEPR* based upon (**a**) BMI and (**b**) Race. The diagonal line is the reference line.

The receiver operating characteristics (ROC) analysis was used to determine the cut-off value for methylation of genes *NRF1*, *FTO*, and *LEPR*. As shown in Table 8, the area under curve (AUC) of PMR of *NRF1*, *FTO*, and *LEPR* with the BMI categories is 0.565 (*p* = 0.236), 0.629 (*p* = 0.018), and 0.571 (*p* = 0.192), respectively. Table 9 shows the ROC analysis of methylation of *NRF1* and *FTO* based on the race, the AUC of *NRF1*, *FTO* and *LEPR* was 0.574 (*p* = 0.179), 0.603 (*p* = 0.059), and 0.850 (*p* = 0.001), respectively. The graphical representation of AUC is shown in Figure 5. As the AUC of *LEPR* is 0.850, it is considered a biomarker with very good diagnostic accuracy. However, the value for *FTO* is between 0.6 and 0.7; it is considered a biomarker with sufficient diagnostic accuracy.

## 4. Discussion

This study explored the differences and the association of methylation of *NRF1*, *FTO*, and *LEPR* genes between normal weight and overweight/obese children taking into consideration of their race, maternal education, and family income. For the genes *NRF1*, *FTO*, and *LEPR*, our study demonstrates a race-specific difference in the DNA methylation of these obesity-related genes. Previously, increased *NRF1* methylation in OW/OB children was also reported by Rushing et al., who found that *NRF1* methylation was significantly associated with a higher probability of childhood obesity [21]. Additionally, DNA methylation and miRNA expression in skeletal muscle samples from sedentary obese and diabetic people, before and after 16 weeks of aerobic or resistance training, showed that *NRF1* methylation was reduced after aerobic exercise [36]. It is important to note here that we used saliva to measure methylation, therefore even though direct comparison cannot be made with studies using blood and skeletal muscle, the fact that we found consistent results opens a window for saliva to be the epigenetic marker, especially for children where collecting tissue and blood is very difficult and painful. Supporting this, Oelsner et al. examined the methylation of obesity-related genes in saliva samples from preschool-age Hispanic children and showed that methylation of one of the CpG sites of *NRF1* was significantly associated with increased BMI [37]. *NRF1* plays a major role as a transcription factor in metabolic regulation and stimulates the expression of *PPARG*, a gene that is highly expressed in adipose tissue controlling numerous genes involved in metabolic homeostasis, lipid, glucose, and energy metabolism, adipogenesis, and inflammation which looks like a promising target and important for future research [38].

Czogala et al. demonstrated higher methylation of gene *FTO* along with increased expression of *FTO* gene in obese children compared to the control group with a significant correlation between *FTO* methylation and gene expression [39]. The gene *FTO* plays a major role in regulating hepatic gluconeogenesis and lipid metabolism, where an increase in *FTO* expression leads to reduced fatty acid oxidation and lipolysis along with increasing triglycerides [40]. In our study, we found that AA normal weight children had almost similar methylation in the *FTO* gene as overweight and obese, signifying a higher gene expression of *FTO* leading them towards increased risk of obesity while *FTO* gene methylation for EA normal weight children was extremely lower. Interestingly, the ROC curve for the gene *FTO* showed statistical significance. However, due to its low prediction capability of <63% of BMI categories correctly, we would suggest further research on *FTO* gene methylation as an early predictor of childhood obesity. Additionally, the results also indicate that in the AA population not only the obesity status, but also other factors such as different health disparities could be playing a role in causing increased DNA methylation, as we did not see a significant association between obesity markers and DNA methylation of *NRF1* and *FTO* gene in AA children. While on the other end, in EA children we saw an increase in DNA methylation of both *NRF1* and *FTO* genes as their BMI *z*-score increased, implying that in the EA population, the methylation status depends more on their obesity status.

To our knowledge, this is the first study demonstrating the differences in *LEPR* methylation between NW and OW/OB children among races. Even though we saw a significant difference in the methylation of the gene, there was no significant association found between methylation and obesity markers. However, in AA children, we could see a significant association between WC *z*-score and *LEPR* methylation after adjusting for maternal education and family income, indicating the important role of health disparities amongst races causing epigenetic modifications. Moreover, AA children generally have higher *LEPR* methylation compared to EA children, signifying that the methylation of *LEPR* in AA does depend more on their BMI status. The leptin receptor protein, which is important in controlling body weight, is made mostly by following the instructions provided by *LEPR.* Through the activation of several signaling pathways, including Janus Kinase 2/Signal transducer and activator of transcription 3 (JAK2/STAT3) and Mitogen Activated Protein Kinase (MAPK cascade), it serves as a receptor for the hormone leptin and mediates *LEP* central and peripheral effects [41]. It is also known that higher body mass is associated with increased leptin hormone in the body. *LEPR* expression is most likely decreased as a result of methylation [42], and therefore higher methylation in normal weight AA could be due to decreased leptin levels as a result of lower body fat. This could be a new window of opportunity to explore whether the obesity associated CpG methylation is caused by obesity, and not causing obesity [43].

Further, obesity prevalence rates differ significantly by race and ethnicity, with African Americans being 50% more likely to be obese than non-Hispanic whites [44], and African American children are at higher risk of being obese [45,46,47]. These results are in consistence with another study, affirming that AA is at a higher risk of obesity [48] and that race, along with the BMI status, is linked with DNA methylation of genes. Epigenetic regulation “levels” differ between racial and ethnic groups, according to research, even after accounting for access to care and genetics, implying that epigenetic regulation may be a contributing reason to health disparities [49]. The age group of 6–10 years can also provide us with an opportunity for early detection of obesity, especially in highly susceptible populations with a potential of methylation of *LEPR* as a biomarker, as the analysis of the ROC curve showed *LEPR* methylation has very good diagnostic accuracy.

Although our study provided a race-specific difference in DNA methylation and a potential link to health disparities, the study’s relatively small sample size may have made it harder to find significant correlations with obesity measures. The results would be strengthened and validated if the current research were to be expanded to cover bigger sample numbers. This study does not consider the genetic effects of these genes on DNA methylation, which can further give more insight on the biological processes. Although there was a precise technique for collecting saliva, there is always a possibility of contamination and human collection error when collecting salivary DNA and doing further clinical procedures. Even though prior research suggests that DNA methylation in saliva and blood samples is similar, the current study only looked at methylation patterns in saliva and cannot be used to directly compare DNA methylation in blood and other tissues. Additionally, this sample provides information on children aged 6–10 years, yet DNA methylation patterns in children of other ages and races/ethnicities should be researched. Further, while collecting the saliva samples, we also recorded the dietary intake of the children, and the association between dietary intake and methylation patterns is underway.

## 5. Conclusions

To conclude, OW/OB children had significantly higher methylation of *NRF1* and *FTO* and lesser methylation of *LEPR*. The methylation of *LEPR* is significantly higher in AA children than EA. Only EA OW/OB children had significantly higher levels of methylation in the *NRF1* and *FTO* genes compared to NW, whereas the AA NW children had higher methylation of the *LEPR* gene than the OW/OB children. After controlling for maternal education and annual family income using regression analysis, the PMR of *NRF1* and *FTO* was significantly associated with BMI *z*-score in EA children but not in AA children. However, the PMR of the gene *LEPR* was significantly associated with all the obesity measures after adjusting the maternal education and family income only in AA children. These findings contribute to a racial difference in the correlation of methylation of the *NRF1*, *FTO,* and *LEPR* genes with childhood obesity.

## Figures and Tables

**Table 1:** Primer and probe sequences (5′ to 3′) for the MethyLight polymerase chain reaction.

Gene	Forward Primer	Reverse Primer	Probe
*NRF1*	GTG GTG GTT TAC GTT TGT AAT TTT AGT	CTA TAT TAA CCA AAA TCG TCT CAA ACT CC	ACC TCA TAT AAT CCG CCT ACC TCG ACC TCC -FAM
*FTO*	GGT TAG TGG TAC GGT GAG TAT TCG	CCA AAA CCT TCT CCA AAC GAC AAA A	AAC CCT AAA ACC CCG ACC CGC GCT ACA AT—ABY
*LEPR*	AGA AAC GGA TTT ACG GAG GAG TTA AGA TGG	TCC CTA ACC CCT TAC GCT TCC CAA A	ACC TCG CCC TAC TTC GAC TCG CAC ACG AT—FAM
*ALU*	GGT TAG GTA TAG TGG TTT ATA TTT GTA ATT TTA GTA	ATT AAC TAA ACT AAT CTT AAA CTC CTA ACC TCA	CCT ACC TTA ACC TCC C—VIC

**Table 2:** General Characteristics of the study population.

Parameter	Total	NW	OW/OB	*p* Value
Number of Participants	113	60	53	-
Sex (male/female)	(58/55)	(32/28)	(26/27)	-
Race (EA/AA)	(63/50)	(31/29)	(32/21)	-
Age (years)	8.57 ± 0.13	8.62 ± 0.19	8.52 ± 0.18	0.701
Height (cm)	133.98 ± 1.10	132.56 ± 1.57	135.58 ± 1.52	0.172
Weight (kg)	34.18 ± 1.05	29.08 ± 0.91	39.97 ± 1.66	**0.001**
BMI (kg/m^2^)	18.63 ± 0.33	16.27 ± 0.21	21.30 ± 0.42	**0.001**
BMI *z*-score	0.97 ± 0.11	0.06 ± 0.11	2.01 ± 0.08	**0.001**
WC (cm)	66.13 ± 13	61.20 ± 0.65	71.72 ± 1.24	**0.001**
WC *z*-score	0.17 ± 0.07	0.21 ± 0.06	1.28 ± 0.07	**0.001**
WHtR *z*-score	0.50 ± 0.07	0.04 ± 0.08	1.05 ± 0.07	**0.001**

NW, Normal weight; OW/OB, Overweight/Obese; EA, European American; AA, African American; BMI, Body Mass Index; WC, Waist circumference; WHtR, weight–height ratio. Data are mean ± SEM. *p* < 0.05 is considered statistically significant between NW and OW/OB participants and it is shown in bold in the table.

**Table 3:** General characteristics for family income and maternal education of the participants.

	All Participants (%)	EA Participants (%)	AA Participants (%)
Family Income			
<USD 25,000	31.9	4.8	66.0
USD 25,000–50,000	13.3	19.0	6.0
USD 50,000–75,000	15.0	20.6	8.0
>USD 75,000	39.8	55.6	20.0
Maternal Education			
High School or less	25.7	9.8	46.0
Associate degree	23.9	17.5	32.0
Bachelor’s degree	18.6	30.2	4.0
Graduate	31.9	42.9	18.0

EA, European American; AA, African American.

**Table 4:** Race-Specific Descriptive Analysis of PMR of *NRF1*, *FTO* and *LEPR*.

Participants	PMR of *NRF1* (%)	*p* Value	PMR of *FTO* (%)	*p* Value	PMR of *LEPR* (%)	*p* Value
All Participants						
NW	68.92 ± 6.45	**0.018**	83.98 ± 17.50	**0.010**	121.40 ± 13.54	**0.025**
OW/OB	102.72 ± 13.03		168.24 ± 28.11		85.07 ± 7.31	
Total	84.77 ± 7.15		123.50 ± 16.54		104.366 ± 8.11	
EA Participants						
NW	48.86 ± 4.25	**0.002**	28.95 ± 1.96	**0.001**	64.07 ± 3.23	0.976
OW/OB	101.67 ± 15.73		177.41 ± 33.61		61.06 ± 3.32	
Total	75.69 ± 8.85		104.36 ± 19.41		62.54 ± 2.30	
AA Participants						
NW	90.36 ± 11.35	0.558	142.80 ± 33.03	0.843	182.69 ± 22.98	**0.046**
OW/OB	104.30 ± 23.00		154.25 ± 50.00		121.67 ± 67.12	
Total	96.21 ± 11.59		147.61 ± 28.12		157.06 ± 15.17	

PMR, Percent Methylation Rate; NW, normal weight; OW/OB, overweight/obese; EA, European American; AA, African American. Data are expressed as mean ± SEM. *p* < 0.05 is considered statistically significant between NW and OW/OB participants and it is shown in bold in the table. An independent sample *t*-test was used for analysis.

**Table 5:** Hierarchical regression analysis of obesity measures and PMR of *NRF1*.

Parameter	Unadjusted				Adjusted			
	β	*p* Value	95% CI		β	*p* Value	95% CI	
			LB	UB			LB	UB
All Participants								
BMI *z*-score	0.089	0.349	−0.002	0.004	0.010	0.914	−0.003	0.003
WC *z*-score	0.116	0.220	−0.001	0.003	0.072	0.453	−0.001	0.002
WHtR *z*-score	0.085	0.369	−0.001	0.003	0.020	0.832	−0.002	0.002
EA Participants								
BMI *z*-score	0.259	**0.040**	0.000	0.009	0.243	**0.037**	0.000	0.009
WC *z*-score	0.242	0.056	0.000	0.005	0.234	0.063	0.000	0.005
WHtR *z*-score	0.228	0.073	0.000	0.005	0.210	0.091	0.000	0.005
AA Participants								
BMI *z*-score	−0.149	0.300	−0.006	0.002	−0.225	0.136	−0.007	0.001
WC *z*-score	−0.043	0.769	−0.003	0.002	−0.068	0.646	−0.003	0.002
WHtR *z*-score	−0.080	0.582	−0.003	0.002	−0.179	0.228	−0.004	0.001

The results were adjusted for annual family income and maternal education. *p* < 0.05 is considered statistically significant between PMR of *NRF1* and obesity markers, and it is shown in bold.

**Table 6:** Hierarchical regression analysis of obesity measures and PMR of *FTO*.

Parameter	Unadjusted				Adjusted			
	β	*p* Value	95% CI		β	*p* Value	95% CI	
			LB	UB			LB	UB
All Participants								
BMI *z*-score	0.171	0.070	0.000	0.002	0.111	0.227	0.000	0.002
WC *z*-score	0.160	0.090	0.000	0.001	0.119	0.209	0.000	0.001
WHtR *z*-score	0.120	0.205	0.000	0.001	0.061	0.509	−0.001	0.001
EA Participants								
BMI *z*-score	0.351	**0.005**	0.001	0.005	0.327	**0.005**	0.001	0.005
WC *z*-score	0.307	**0.014**	0.000	0.003	0.297	**0.019**	0.000	0.003
WHtR *z*-score	0.278	**0.027**	0.000	0.003	0.253	**0.044**	0.000	0.003
AA Participants								
BMI *z*-score	−0.047	0.746	−0.002	0.001	−0.119	0.411	−0.002	0.001
WC *z*-score	−0.002	0.990	−0.001	0.001	−0.070	0.621	−0.001	0.001
WHtR *z*-score	−0.042	0.771	−0.001	0.001	−0.135	0.340	−0.002	0.001

The results were adjusted for annual family income and mother’s education. *p* < 0.05 is considered statistically significant between PMR of *FTO* and obesity markers and it is shown in bold.

**Table 7:** Hierarchical regression analysis of obesity measures and PMR of *LEPR*.

Parameter	Unadjusted				Adjusted			
	β	*p* Value	95% CI		β	*p* Value	95% CI	
			LB	UB			LB	UB
All Participants								
BMI *z*-score	−0.095	0.315	−20.057	6.527	−0.084	0.385	−19.622	7.635
WC *z*-score	−0.112	0.236	−35.665	8.874	−0.084	0.372	−32.240	12.155
WHtR *z*-score	−0.154	0.103	−38.819	3.600	−0.126	0.188	−36.008	7.137
EA Participants								
BMI *z*-score	−0.018	0.886	−3.868	3.350	−0.065	0.652	−4.932	3.109
WC *z*-score	−0.130	0.310	−9.506	3.069	−0.166	0.209	−10.596	2.362
WHtR *z*-score	−0.016	0.901	−6.427	5.672	−0.051	0.703	−7.533	5.113
AA Participants								
BMI *z*-score	−0.280	**0.049**	−54.277	−0.124	−0.317	**0.035**	−59.380	−2.260
WC *z*-score	−0.257	0.072	−81.466	3.592	−2.134	**0.038**	−94.743	−2.770
WHtR *z*-score	−0.306	**0.031**	−84.535	−4.327	−0.348	**0.023**	−93.708	−7.458

The results were adjusted for annual family income and the mother’s education. *p* < 0.05 is considered statistically significant between PMR of *LEPR* and obesity markers and it is shown in bold.

**Table 8:** ROC curve analysis of methylation of *NRF1, FTO,* and *LEPR* with the BMI category shows the relationship between sensitivity and specificity in determining a specific marker.

Parameter	AUC	SE	Cut-Off	Sensitivity	1-Specificity	*p* Value	95% CI	
							LB	UB
*NRF1*	0.565	0.056	121.089	0.377	0.117	0.236	0.455	0.674
*FTO*	0.629	0.052	37.635	0.547	0.350	**0.018**	0.526	0.732
*LEPR*	0.571	0.054	60.64	0.700	0.623	0.192	0.466	0.677

*p* < 0.05 is considered statistically significant and it is shown bold in the table. ROC—receiver operating curve; AUC—area under curve; SE—standard error; CI—confidence interval.

**Table 9:** ROC curve analysis of methylation of *NRF1, FTO,* and *LEPR* with the races shows the relationship between sensitivity and specificity in determining a specific marker.

Parameter	AUC	SE	Cut-Off	Sensitivity	1-Specificity	*p* Value	95% CI	
							LB	UB
*NRF1*	0.574	0.056	64.220	0.560	0.333	0.179	0.465	0.683
*FTO*	0.603	0.053	35.444	0.600	0.413	0.059	0.500	0.707
*LEPR*	0.850	0.036	67.305	0.820	0.286	**0.001**	0.780	0.920

*p* < 0.05 is considered statistically significant and it is shown bold in the table. ROC—receiver operating curve; AUC—area under curve; SE—standard error; CI—confidence interval.

## Data Availability

The study datasets of the current manuscript are available from the corresponding author upon request.
